# A Comparative Biomechanical Study of Alternative Medial Collateral Ligament Reconstruction Techniques

**DOI:** 10.1177/03635465241235858

**Published:** 2024-03-29

**Authors:** Jobe Shatrov, Petra Bonacic Bartolin, Sander R. Holthof, Simon Ball, Andy Williams, Andrew A. Amis

**Affiliations:** * Imperial College London, London, United Kingdom; † Fortius Clinic, London, United Kingdom; Investigation performed at Imperial College London, London, United Kingdom

**Keywords:** medial collateral ligament, reconstruction techniques, biomechanical testing, anteromedial rotatory instability

## Abstract

**Background:**

There is little evidence of the biomechanical performance of medial collateral ligament (MCL) reconstructions for restoring stability to the MCL-deficient knee regarding valgus, external rotation (ER), and anteromedial rotatory instability (AMRI).

**Hypothesis:**

A short isometric reconstruction will better restore stability than a longer superficial MCL (sMCL) reconstruction, and an additional deep MCL (dMCL) graft will better control ER and AMRI than single-strand reconstructions.

**Study Design:**

Controlled laboratory study.

**Methods:**

Nine cadaveric human knees were tested in a kinematics rig that allowed tibial loading while the knee was flexed-extended 0° to 100°. Optical markers were placed on the femur and tibia and displacements were measured using a stereo camera system. The knee was tested intact, and then after MCL (sMCL + dMCL) transection, and loaded in anterior tibial translation (ATT), ER, varus-valgus, and combined ATT + ER (AMRI loading). Five different isometric MCL reconstructions were tested: isolated long sMCL, a short construct, each with and without dMCL addition, and isolated dMCL reconstruction, using an 8 mm–wide synthetic graft.

**Results:**

MCL deficiency caused an increase in ER of 4° at 0° of flexion (*P* = .271) up to 14° at 100° of flexion (*P* = .002), and valgus laxity increased by 5° to 8° between 0° and 100° of flexion (*P* < .024 at 0°-90°). ATT did not increase significantly in isolated MCL deficiency (*P* > .999). All 5 reconstructions restored native stability across the arc of flexion apart from the isolated long sMCL, which demonstrated residual ER instability (*P*≤ .047 vs other reconstructions).

**Conclusion:**

All tested techniques apart from the isolated long sMCL graft are satisfactory in the context of restoring the valgus, ER, and AMRI stability to the MCL-deficient knee in a cadaveric model.

**Clinical Relevance:**

Contemporary MCL reconstruction techniques fail to control ER and therefore AMRI as they use a long sMCL graft and do not address the dMCL. This study compares 5 MCL reconstruction techniques. Both long and short isometric constructs other than the long sMCL achieved native stability in valgus and ER/AMRI. Double-strand reconstructions (sMCL + dMCL) tended to provide more stability. This study shows which reconstructions demonstrate the best biomechanical performance, informs surgical reconstruction techniques for AMRI, and questions the efficacy of current popular techniques.

Overall, 60% of anterior cruciate ligament (ACL) injuries are combined with medial collateral ligament (MCL) injuries.^32^ Because MCL deficiency increases tension in the ACL during rotational movements, care must be taken to treat the MCL after injury occurs.^6,9,28^ An MCL tear can be treated surgically or nonsurgically.^10,27^ However, nonsurgical treatment of a concomitant MCL injury without adequate healing in the setting of ACL reconstruction increases the risk of graft failure and chronic symptoms when the knee is loaded in external rotation (ER), especially in the sports-active population.^2,3,26,30^ Of course, in isolated MCL injuries, there is also the risk of symptoms from excessive MCL laxity if inadequate healing occurs. Some high-grade isolated MCL injuries do need primary surgical intervention.

Although the importance of the ACL for knee stability has been studied extensively, the role of the MCL in isolated and combined injuries has been neglected.^31^ The MCL helps to protect the ACL from injury.^4^ The primary medial soft tissue restraint of ER of the tibia is the deep band of the MCL (dMCL) in 0° to 30°, while the superficial band (sMCL) is the main restraint of valgus and of ER in deeper flexion.^7^ Injuries that impose excessive ER torque on the tibia, causing anteromedial rotatory instability (AMRI), first tear the dMCL, then the sMCL, and finally the ACL.^18,29^ Neglecting effective treatment of the MCL in such combined ACL/MCL injuries results in approximately two-thirds of injuries with persistent knee laxity and AMRI.^1^ Clinical data show increased ACL graft rerupture rates in the presence of even minor unaddressed MCL laxity.^2,30^ In 1 publication, the increased risk of ACL rerupture was stated to be 17-fold.^3^

Despite the importance of effective concomitant MCL treatment being highlighted in these studies,^2,3,30^ it is still unknown which MCL reconstruction method is best for stabilizing both ER/AMRI and valgus instabilities.^34^ An sMCL graft placed isometrically on the medial epicondyle restores valgus stability through the knee flexion arc.^24,25^ However, a recent cadaveric study concluded that AMRI caused by an injured sMCL and dMCL complex, plus ACL rupture, could not be abolished by isolated sMCL reconstruction, but was abolished by adding an oblique dMCL graft.^8,9^ Both double- and triple-strand constructs that included an anatomically placed dMCL graft could restore native ER and abolish AMRI.^24,25^

It is usually attractive to reduce the complexity and invasiveness of surgical procedures, and that approach may be applied to MCL reconstructions. A potential problem with anatomic placement of both sMCL and dMCL grafts is conflict of the femoral tunnels because their femoral attachments are close together.^5^ A single femoral tunnel for both grafts would simplify the procedure and avoid tunnel conflict or convergence. The similar orientations of the dMCL and sMCL raise the possibility of combining them into a single-strand construct oriented between the 2 native structures. However, that would leave the graft with less ideal orientation to resist both valgus and ER loading. Additionally, the sMCL attaches 60 mm below the joint line,^5^ raising the possibility of reducing invasiveness by moving the tibial attachment proximally, allowing a shorter wound, better bone for graft fixation, and less risk of graft/implant irritation of the pes anserine tendons. These considerations led to the development of a short isometric construct attaching 20 to 30 mm below the joint line and at the anterior edge of the sMCL,^11^ but its ability to stabilize AMRI has not been examined. Previous studies have used different constructs, bone attachments, and graft tensioning protocols. Therefore, there is a need for further development and testing of MCL reconstructions to simplify the procedure yet ensure that knee stability will be restored.

The purpose of this study was to compare a range of possible MCL graft configurations that approximate anatomic structures: the sMCL reconstruction and the short construct, each with or without the addition of an oblique dMCL graft, and an isolated dMCL reconstruction. It was hypothesized that the short reconstruction would better restore valgus and ER stability compared with a longer sMCL reconstruction, and that with an additional dMCL graft, the resulting double-strand reconstructions would better control ER and AMRI than single-strand reconstructions.

## Methods

### Specimen Preparation

Nine fresh-frozen cadaveric human knees, from 3 male and 4 female donors (mean age, 48 years; range, 27-59 years), were obtained from MedCure with institutional review board approval (Imperial College Healthcare Tissue Bank Project R18027-5A). Before preparation, all samples were stored at −20°C and defrosted for 24 hours at room temperature before use. Based on visual and physical examination by an orthopaedic surgeon (J.S.), the knees showed no signs of previous surgery, abnormal laxity, or malalignment.

**Figure 1.**
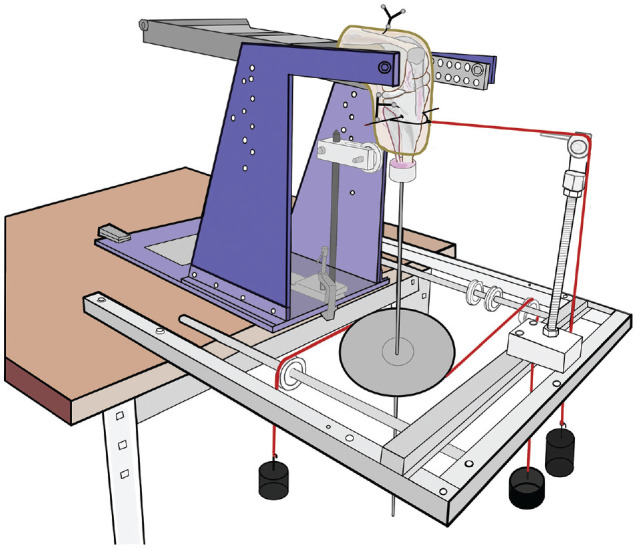
Kinematics rig with reflective marker arrays mounted on the femur and tibia for optical tracking. The 2 hanging weights attached to the central pulley induce tibial rotation, and the weight and pulley system attached to the proximal tibia induces anterior tibial translation. Raising the frame with the femur mounted on it causes the knee to extend.

The skin and subcutaneous fat were removed with care so no damage occurred to the deeper soft tissue. The femur was cut to 170 mm above the joint line, and the tibia to 120 mm below. All soft tissue >70 mm from the joint line was removed. The proximal fibula was transected at 100 mm length and stabilized on the tibia in the native position with a screw. The femoral fixation on the kinematics rig used a cylindrical stainless steel pot, while the distal tibia required a pot with a rod extending 0.5 m from its distal end to allow loads to be applied. The tibia hung vertically below the distal femur, which was secured to the moving arm of a 6 degrees of freedom kinematics rig with the femoral shaft at the anatomic 6° of valgus offset.^17,21^ The femoral position was then adjusted so that the tibia hung vertically at 0° and 90° of flexion and the transepicondylar axis was along the flexion-extension axis of the test rig (Figure 1). During the alignment process the bones were secured in their pots using sharp-pointed screws; then finally, polymethyl methacrylate bone cement was added to the pots to achieve secure bone fixation. A clamp on the rod extending from the tibia allowed it to be secured in neutral internal rotation (IR)–ER at 30° of flexion. This reproducible position was used when tensing the MCL grafts.^17^

After placement of the specimen in the kinematics rig, 3 digitizing screws were placed into both the tibia and femur. The tibial screws were at the most prominent medial and lateral points near the tibial plateau, plus a distal anterior screw. The femoral medial and lateral digitizing screws were placed 10 mm proximal to the epicondyles to protect the collateral ligament attachments and avoid the MCL graft tunnel, plus a proximal screw. These screws defined the coordinate system during kinematic measurement.

### Knee Loading Parameters

Kinematic parameters of anterior tibial translation (ATT) and posterior tibial translation, ER-IR, valgus, and varus rotation, and combined ATT + ER movement (AMRI loading), were monitored.^24,25^ Tibial ATT/posterior tibial translation displacing forces were imposed using 88-N hanging weights with ropes led over pulleys to 2 semicircular metal hoops mounted around the tibia using a transverse Steinmann pin 40 mm below the joint line. The load levels were based on data from clinical examinations.^4,18,34^ This mechanism allowed the transmission of anteroposterior forces without inhibition of IR-ER. The tibia was loaded to 5 N·m IR-ER torque using a 250-mm pulley fixed on the distal tibial extension rod, with hanging weights. Similarly, varus-valgus moments of 8 N·m were imposed by pulling medially and laterally at the distal end of the rod, again with hanging weights and pulleys (Figure 1).^17,21^

### Measurement of Knee Stability

A stereo optical measuring system with an accuracy of ±0.12 mm^19^ (Polaris Vega; Northern Digital Inc) measured the 3-dimensional positions of passive marker arrays attached to the femur and tibia with bicortical rods. To define the neutral (unloaded) path of movement of the native knee, 10 flexion-extension cycles from 0° to 100° were measured with the tibia unconstrained and the mean values calculated. The neutral position of tibial IR-ER was recorded on the test rig at 30° of flexion, which was the posture to be used when tensioning the grafts later. Further kinematic data were collected during 3 cycles of flexion-extension. Custom code (MATLAB; MathWorks) was used to transform the raw data into a clinical description of the 6 degrees of freedom knee joint kinematics. All subsequent measures were expressed in relation to the neutral path of motion datum: laxity was defined as the change of motion when load was applied to the native knee, and instability was defined as the increase of laxity beyond that of the native knee after ligament transection and reconstruction. Kinematics were measured first on the native sample and then with the MCL cut (sMCL + dMCL both transected) and with 5 different types of MCL reconstructions. Zero degrees of flexion was defined as when the tibial and femoral rods were parallel when viewed in the sagittal plane.^21,25^

### MCL Reconstruction Techniques

**Figure 2.**
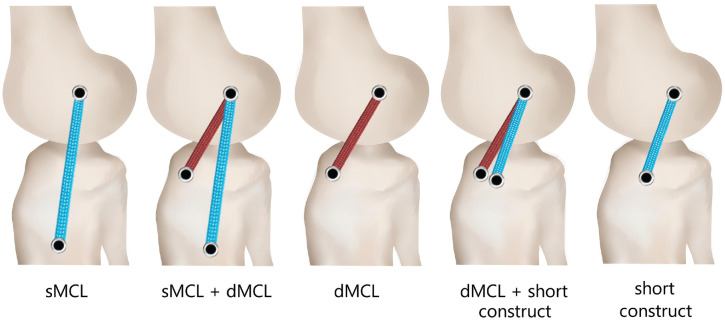
Medial collateral ligament (MCL) reconstruction techniques: isolated superficial MCL (sMCL), sMCL + deep MCL (dMCL), isolated dMCL, short construct + dMCL, and isolated short construct.

Five different medial reconstructions were used, covering single and double strands and long and short grafts, all based on a single isometric femoral tunnel at the medial epicondyle. The tests were performed in the order shown to minimize intervention steps such as releasing and later retensioning structures (Figure 2):

- Isolated sMCL (long)
- sMCL + dMCL
- Isolated dMCL
- Short construct + dMCL
- Isolated short construct

For all reconstructions, an 8 mm–wide braided polyethylene implant (Dynatec Sling; Climbers-Shop.com) was used to simulate a clinical graft, with No. 2 sutures (Ultrabraid; Smith & Nephew) secured into its ends.

For each reconstruction, the isometric position of the femoral tunnel was determined by placing 2.4-mm surgical wires at the chosen anatomic landmarks for graft placement.^5^ The femoral wire was initially placed at the isometric point of the femoral sMCL insertion, 1 mm proximal to the medial epicondyle, which is centered at 47% of the anteroposterior size of the medial femoral condyle anterior from the most posterior limit and 47% proximal from the most distal limit.^5^ For tibial fixation, for the anatomic (long) sMCL reconstruction, the guide wire was placed 60 mm below the plateau at the midwidth of the native attachment, while for the short (nonanatomic) construct, the tibial wire was placed 20 mm below the plateau and at the midpoint of the anterior half of the width of the native sMCL, giving a slightly oblique graft.^11^ The isometry of the planned construct was determined using a suture led from the femoral wire to the tibial wire, and the femoral position was adjusted as necessary to ensure isometry. If the suture lengthened with knee flexion, the femoral pin was anterior to the isometric point, and vice versa. Then a 4.5-mm bicortical tunnel was drilled in the femur, followed by a 7-mm socket drilled to but not through the lateral cortex. For the femur, lead sutures were tied over a cortical button (Endobutton; Smith & Nephew) on the lateral cortex. A 7 × 25–mm interference screw (RCI; Smith & Nephew) was used for additional interference fixation at the femoral tunnel aperture to reduce the risk of graft slippage through repeated load cycles. The dMCL graft was fixed into the same femoral tunnel and, with the knee held at 15° of flexion and neutral tibial rotation, passed 25° anterodistally to a tunnel 10 mm below the tibial plateau and just anterior to the sMCL on the tibia.^5^ Each graft was tensioned and fixed into a 7-mm bicortical tibial tunnel at 30° of flexion with the tibia clamped in the previously defined neutral rotation. After temporary fixation, the knee was flexed-extended 15 times, and then the distal end of the graft was secured while tensioned through the tibial tunnel using a tensiometer to 60 N for the sMCL and short construct and 20 N for the dMCL, with the tibia loaded into 2 N·m varus to reduce the medial compartment. These tensions followed previous work showing that MCL reconstructions using them restored native stability.^24,25^ The grafts were fixed using a 7 × 25–mm interference screw (RCI; Smith & Nephew) in the tibial tunnel entrance, plus a screw post for the lead sutures at the lateral aspect. The dMCL graft was placed in the distal part of the femoral tunnel entrance with the sMCL/short construct graft more proximal, so that when passed toward their tibial attachments the dMCL graft was deep to the other graft (Figure 2). No graft retensioning was needed because of the high strength and creep resistance of the 8-mm tape used; no tape slippage from the fixations was found by visual inspection after marking the tapes and tunnel entrances with ink lines.

### Statistical Analysis

The number of samples required was calculated by power analysis with G*Power Version 3.1.9.7 (Heinrich Heine University) using published data,^13^ which revealed that a change of 2° of ER could be identified with 88% power and 95% confidence with 7 specimens. Nine knees were used in this research because we did not anticipate our data to be as consistent as the published data.

The Shapiro-Wilk test was used to check that the data distributions were compatible with parametric analysis. Statistical analysis used a 2-way analysis of variance with repeated measures to determine whether there were significant differences between groups (SPSS Statistics software; IBM Corp). Key variables were knee treatment type (intact, injured, or MCL reconstruction) and knee flexion angle, and differences between treatment conditions were examined for each laxity measurement. Changes in knee laxity were the dependent variables. With Bonferroni adjustment for multiple contrasts, repeated-measures *t* tests at each 10° of knee flexion were used for post hoc testing if significant effects were detected. *P* < .05 indicates significance.

## Results

### Anterior Translation

Transection of the MCL did not cause any significant increase of ATT across 0° to 100° of flexion (*P* > .999), and similarly there were no significant differences among the reconstructions.

### External Rotation

**Figure 3.**
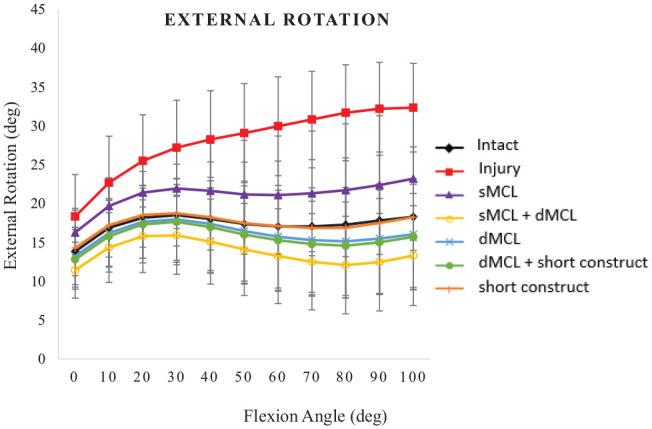
External rotation (deg) under a torque of 5 N·m for the following cases: intact medial collateral ligament (MCL), injury (superficial MCL [sMCL] and deep MCL [dMCL] transected), sMCL reconstruction, sMCL + dMCL reconstruction, dMCL reconstruction, dMCL + short construct, and short construct. Data are presented as mean ± SD for 9 knees.

MCL deficiency (both sMCL and dMCL transected) caused ER to increase by 4° at 0° of flexion (*P* = .271) and up to 14° at 100° of flexion (*P* = .002), with significant instability observed between 20° and 100° (Figure 3).

The sMCL graft did not reduce ER significantly from that of the MCL-deficient knee across 0° to 100° of flexion (*P*≥ .450). It was significantly more unstable than the short construct + dMCL, isolated dMCL, sMCL + dMCL, and isolated short construct reconstructions from 90° to 100° of flexion (*P*≤ .047). All the other reconstructions, including the isolated dMCL and short isometric constructs, reduced ER such that it did not differ significantly from native ER (MCL intact) across 0° to 100° of flexion. Significant differences were not found among the other 4 reconstructions, but the double-strand reconstructions tended to be more stable in ER with the graft tensions used.

### Valgus

**Figure 4.**
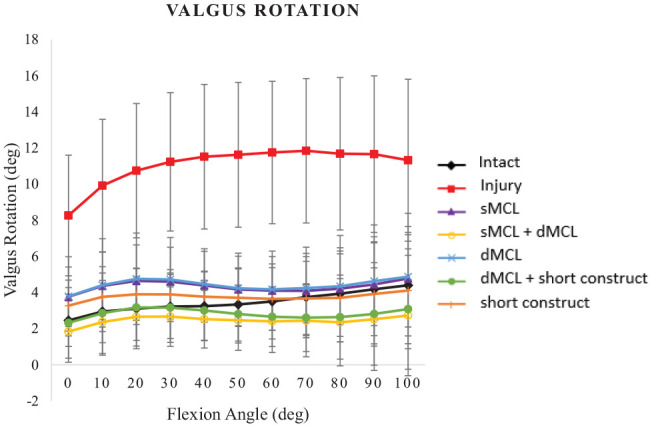
Valgus rotation (deg) under a moment of 8 N·m for the following cases: intact medial collateral ligament (MCL), injury (superficial MCL [sMCL] and deep MCL [dMCL] transected), sMCL reconstruction, sMCL + dMCL reconstruction, dMCL reconstruction, dMCL + short construct, and short construct. Data are presented as mean ± SD for 9 knees.

MCL transection caused valgus instability to increase by 0° to 8° across 0° to 100° of flexion, which was significant (*P* < .024) across 0° to 90° of flexion, but not (*P* = .086) at 100° (Figure 4).

All reconstructions restored valgus such that it did not differ from intact stability across 0° to 100° of flexion (*P*≥ .533). When comparing the reconstructions in resisting valgus, there was no difference between them from 10° to 30° of flexion (*P*≥ .121), while the short construct + dMCL and sMCL + dMCL were both more stable than the dMCL alone from 50° to 90° of flexion (*P*≤ .041). They were not significantly different from the isolated short construct.

### Varus Rotation

Transecting and reconstructing the MCL did not affect varus laxity significantly, with all measurements being within 1° at each angle of flexion across 0° to 100° of flexion.

### Combined Anterior Translation Plus ER (AMRI Test)

#### ATT in Response to AMRI Loading

**Figure 5.**
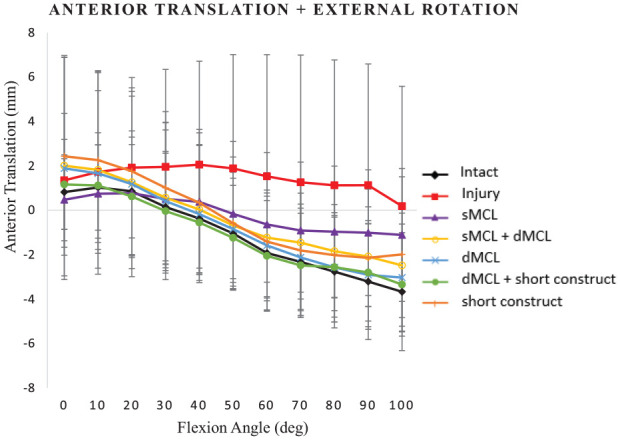
Anterior tibial translation (mm) in response to anteromedial rotatory instability loading (combined 90-N anterior translation force and 5-N·m external rotation torque) for the following cases: intact medial collateral ligament (MCL), injury (superficial MCL [sMCL] and deep MCL [dMCL] transected), sMCL reconstruction, sMCL + dMCL reconstruction, dMCL reconstruction, dMCL + short construct, and short construct. Data are presented as mean ± SD for 9 knees.

The increase of ATT after MCL transection of approximately 4 mm in flexion was not significant (*P*≥ .363) (Figure 5).

The ATT in response to AMRI testing did not differ significantly from native stability for any of the reconstructions at any flexion angle (*P* > .999). Thus, MCL transection and reconstruction did not alter ATT in the presence of the intact ACL.

#### External Rotation in response to AMRI loading

**Figure 6.**
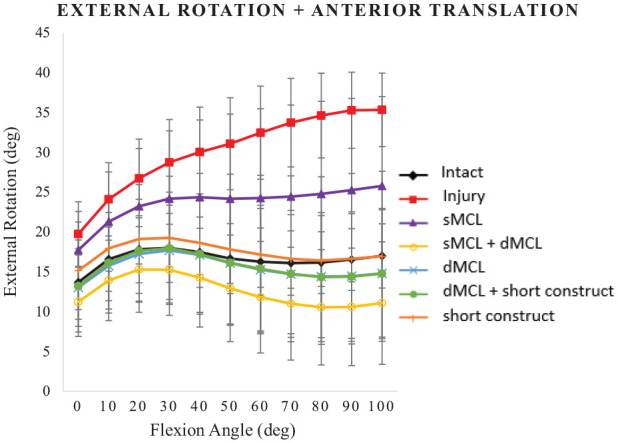
External rotation (deg) in response to anteromedial rotatory instability testing. Combined 90-N anterior translation force and 5-N·m external rotation torque for the following cases: intact medial collateral ligament (MCL), injury (superficial MCL [sMCL] and deep MCL [dMCL] transected), sMCL reconstruction, sMCL + dMCL reconstruction, dMCL reconstruction, dMCL + short construct, and short construct. Data are presented as mean ± SD for 9 knees.

Under AMRI loading, MCL transection caused ER to increase significantly across 0° to 100° of flexion (*P*≤ .003), reaching 35° of ER at 90° and 100° of flexion (Figure 6).

The isolated sMCL reconstruction did not reduce ER in response to AMRI testing significantly below that of the injured knee (*P*≥ .153). Only the short construct + dMCL reconstruction reduced ER significantly at 0° of flexion (*P* = .011), and all reconstructions, other than the isolated sMCL, reduced ER from 10° to 100° of flexion (*P*≤ .041 at 10° to *P* < .001 at 90°).

The ER stability in response to AMRI loading after MCL reconstruction did not differ significantly from native ER stability for all reconstructions, apart from the isolated sMCL, which failed to control it across 0° to 100° of flexion (*P*≥ .123). Comparing reconstructions, the isolated long sMCL reconstruction allowed residual ER instability versus all the other reconstructions across 0° to 100° of flexion, other than the short construct that did not differ significantly from the sMCL reconstruction across 0° to 30° of flexion. The double-strand reconstructions were significantly more stable than the isolated sMCL reconstruction (*P* < .011 for short construct + dMCL and *P*≤ .039 for sMCL + dMCL).

## Discussion

This biomechanical study has shown that all the MCL reconstructions studied, apart from the long isolated sMCL construct, could restore both valgus stability and ER stability, matching the native knee across 0° to 100° of flexion under the chosen loads. Comparison among the reconstructions found that, as hypothesized, the long isolated sMCL reconstruction was significantly less stable than the other constructs in ER, while the double-strand reconstructions were significantly more stable than the long sMCL in ER under AMRI loading. Contrary to the hypothesis, the short isolated single-strand reconstructions (short sMCL and dMCL) were not significantly inferior to the double-strand reconstructions. These results depended on several factors, including use of a synthetic 8-mm flat tape graft with consistent resistance to length increase, appropriate graft tensioning, and an isometric graft tunnel placed at the medial epicondyle.

The motivation for this study was the evidence that persisting MCL deficiency leads to a large increase in failure of ACL reconstructions in combined injuries,^2,3,30^ but there has been little objective evidence to compare MCL reconstructions. Although it has long been known that the sMCL is the primary restraint of valgus,^16^ it has only recently been shown that the deep band (dMCL) is the primary restraint of ER in the 0° to 30° range.^7^ Kennedy and Fowler^18^ and Slocum and Larson^29^ found that dMCL failure is “the essential lesion” in AMRI, and most ACL injuries in soccer occur with a combined valgus plus ER mechanism.^15^ Many injuries treated as being an isolated ACL rupture have later been found to have associated MCL lesions.^32^ These findings support the development of MCL reconstructions that take into account the dMCL as well as the sMCL.

The traditional MCL reconstruction has been a single sMCL graft passing from the femoral medial epicondylar region down to a tibial attachment, typically 60 mm below the joint line.^5^ The epicondyle is the isometric point,^33^ as used for the femoral graft tunnels in the present study. An alternative position proposed in 1 study, posterior to the epicondyle,^13^ causes the MCL graft to slacken in knee flexion, allowing residual instability.^25^ In contrast, isometric grafts restore native valgus stability across the arc of flexion, as in the present study and previously.^24,25^

In the present study, the long sMCL graft failed to control ER. Fixation distal on the tibia requires considerable ER before it attains sufficient obliquity to resist rotation. In an earlier technique,^13^ additional suture fixation close to the tibial plateau was undertaken. However, this technique failed to control ER in a previous cadaveric study.^25^

An oblique graft orientation will help to stabilize ER. A hamstring tendon graft detached proximally but left attached distally has an oblique orientation when fixed to the medial epicondyle and could be advantageous.^23^ A dMCL reconstruction using a doubled gracilis tendon graft was described as a “mirror of an anterolateral ligament reconstruction.”^14^ A similar short isometric construct used a synthetic graft passing from the medial epicondyle to a point 20 mm below the tibial plateau and at the midpoint of the anterior half of the width of the native sMCL, giving a slightly oblique graft.^11^

Studies of double-strand (dMCL + sMCL) or triple-strand (dMCL + sMCL + posterior oblique ligament [POL]) reconstructions have found that an additional oblique dMCL graft improves ER and AMRI stability.^9,24,25^ However, it remains difficult to choose the best option, given the different grafts, tunnel positions, tensioning, and loading parameters among the studies, as well as the choice of leaving the ACL intact (which can be considered as being a perfect ACL reconstruction) or reconstructed (which adds further variability). Thus, the present study has compared single- and double-strand reconstructions and shorter, less invasive procedures with the tibial sMCL attachment 20 mm below the joint line. Although the native dMCL is not the primary restraint of valgus at any angle of knee flexion, nor of ER in the flexed knee, the present study shows that, in the absence of the sMCL, a tensioned isometric dMCL or short isometric construct graft can restore native valgus, ER, and AMRI stability. However, it is uncertain how this may translate into clinical outcomes because the tests were at comparatively low loads and were not cyclic. Furthermore, the oblique grafts were not aligned optimally to resist valgus loading. Overall, the double-strand reconstructions tended to provide better stability, and it could be speculated that they might resist cyclic load elongation better than single-strand oblique constructs. The native dMCL attaches approximately 6 mm distal and posterior to the epicondyle.^5^ This proximity suggests that separate sMCL and dMCL graft tunnels may not be needed; the present study shows that to be the case. This avoids the risk of tunnel conflict and the cost of extra fixation devices. Similarly, Zhu et al^34^ combined the sMCL and POL grafts into a single femoral tunnel to restore IR stability.

The medial soft tissues have a complex structure, so it is difficult to restore their function completely with reconstruction alone. The senior surgeon authors (S.V.B. and A.W.) repair these tissues in the acute setting and use sutures to take up slack in chronic cases, thus restoring native anatomy. However, a reconstruction is also undertaken to protect the repairs in the short term until healing is complete.^11^ This concept leads to better results than soft tissue repair alone in posterolateral ligament complex surgery.^22^

The choice of graft for MCL reconstruction is complicated by medial soft tissue damage, as harvesting a hamstring tendon graft will worsen the medial deficit through weakened dynamic control against valgus and ER. An alternative is using contralateral hamstring grafts. The present study used an 8 mm–wide synthetic graft to ensure reproducibility between the 5 reconstructions tested; its flat cross section was more like the native ligament than a round tendon or cord would be, providing better stability.^1,9^ The synthetic graft did not suffer the degradation and stretching out that would have affected an autograft or allograft tendon across 5 reconstructions. Although not a clinical product, it was ideal in the experimental setting in the laboratory. The surgeon authors use similar medical devices in their clinical practice.

Synthetic grafts in knee ligament surgery are controversial, with worries regarding adverse biological reactions and graft failure. The surgeon authors of the present study found that soft tissue grafts may stretch excessively in MCL reconstruction, so they have used synthetic grafts to augment medial soft tissue repairs for many years.^11^ This method was used successfully in 68 multiligament injury cases in elite sports, with 48 combined ACL + MCL reconstructions.^12^ Of these, 43 (90%) of the patients returned to play, 41 at the same or higher level compared with their preinjury status, at a mean of 10.9 months. No adverse biological reactions occurred. A further study reported 76 cases of synthetic grafts used in collateral ligament reconstructions in elite athletes, with 64 MCL cases.^6^ Overall, 88% returned to play, with 97% at the same or higher level. Overall, 84% and 57% were playing at 2 and 5 years, respectively. One MCL reruptured at 4 years. One MCL graft was removed because of pain from an inflammatory reaction, but the failure mechanism is unknown. It seems that extra-articular use of a synthetic graft is efficacious and safe.

Synthetic grafts may cause overconstraint if they have nonphysiological high graft stiffness in combination with nonisometric placement and inappropriate tensioning. Therefore, if using a synthetic graft, it is critical to avoid a nonisometric femoral tunnel and not to overtension the graft. Graft isometry is most sensitive to the position of the femoral tunnel.^20^ The use of 60 N for the sMCL and short construct grafts and 20 N for the dMCL graft followed previous work showing that reconstructions using these tensions restored native stability.^24,25^ Importantly, the tibia was held in neutral rotation when the grafts were tensioned. Work on lateral extra-articular tenodesis showed that this method avoided capturing the knee in excess ER.^17^

### Limitations

This work used cadaveric tissue, so it could not account for tissue-healing effects on the graft and fixation and therefore only represented a time-zero state. It could only evaluate the stability resulting from the passive ligaments and their reconstructions and not the effects of muscle loading or sports activities. It is also possible that the results may have been affected by using synthetic grafts rather than autogenous tendons, which might be subject to greater slipping and creep elongation when loaded. A more complete evaluation might have added cyclic loading to simulate loads imposed during rehabilitation in vivo; it could be speculated that the double-strand reconstructions would then suffer less elongation than a single-strand reconstruction. The loads used were in line with many other studies simulating clinical examination of knee stability. The specimens available were older than the usual age when sports injuries occur and would have had reduced tissue properties, so the reconstructions used backup fixations to reduce graft slippage under load. The ACL was left intact and was considered as an ideal ACL reconstruction, so this work could not determine how an ACL reconstruction would affect the stability, but this approach meant that variability among ACL reconstructions did not mask the differences sought among the MCL reconstructions. Additional transection and reconstruction of the ACL might add larger changes of ATT and increase ER instability. Finally, this work only studied the 2 bands of the MCL, and in some circumstances the injury pattern might additionally affect structures such as the POL, medial meniscus, and anteromedial retinaculum. A POL reconstruction is not usually indicated because of its posterior orientation, which causes it to slacken with ER, and its presence or absence has no effect on AMRI laxity.^25^ These limitations are countered by the ability to study repeated procedures in each knee, to be able to instrument the knees and apply known loads while measuring stability accurately, and then the use of repeated-measures statistical analysis, which eliminates the masking effects of interspecimen variability. Although the findings of this biomechanical study are clear, translation into clinical evaluation requires care.

## Conclusion

All 5 MCL reconstructions, which were based on an isometric femoral graft tunnel, restored native valgus stability across 0° to 100° of flexion in a cadaveric model. An isolated long sMCL graft did not reduce ER instability significantly compared with the injured knee, while the double-strand (sMCL + dMCL) reconstructions were significantly more stable than the isolated long sMCL graft. The isolated dMCL and short construct grafts, with their oblique orientations, each fully restored ER stability. The overall picture was that, even though all reconstructions other than the isolated long sMCL were capable of restoring ER stability both in isolated ER loading and as part of AMRI loading, the double-strand constructs tended to provide greater ER and valgus stability. These findings have implications for surgical reconstruction techniques that aim to address AMRI in acute and chronic settings as well as MCL reconstruction generally.
